# Probing the folding of mini-protein Beta3s by two-dimensional infrared spectroscopy; simulation study

**DOI:** 10.1186/1757-5036-3-8

**Published:** 2010-03-19

**Authors:** Christopher NJ Marai, Shaul Mukamel, Jin Wang

**Affiliations:** 1 Graduate Program in Biochemistry and Structural Biology, State University of New York at Stony Brook, New York, 11794-3400, USA; 2 Department of Chemistry, University of California, Irvine, CA, 92697-2025, USA; 3 Department of Chemistry, State University of New York at Stony Brook, New York, 11794-3400, USA; 4 Department of Physics, State University of New York at Stony Brook, New York, 11794-3400, USA

## Abstract

We propose to use infrared coherent two-dimensional correlation spectroscopy (2DCS) to characterize the folding mechanism of the mini-protein Beta3s. In this study Beta3s was folded by molecular dynamics (MD) simulation and intermediate conformational ensembles were identified. The one and two-dimensional correlation spectrum was calculated for the intermediate and native states of the mini-protein. A direct structure-spectra relationship was determined by analysis of conformational properties and specific residue contributions. We identified the structural origin of diagonal and off-diagonal peaks in the 2DCS spectra for the native and intermediate conformational ensembles in the folding mechanism. This work supports the implementation of computational techniques in conjunction with experimental 2DCS to study the folding mechanism of proteins. In addition to exploring the folding mechanism the work presented here can be applied in combination with experiment to refine and validate current molecular dynamics force fields.

**PACS Codes: **87.15.Cc, 87.15.hm, 87.15.hp

## 1. Introduction

The biological activities of proteins are determined by the specific three-dimensional structure and dynamical properties of the molecule. The activity of misfolded proteins has been implicated in diseases including Alzheimer's, Diabetes, Parkinson's disease, many cancers and cancer-related syndromes, consequently an understanding of the protein folding mechanism is of importance to pharmaceutical design and molecular biology [1-5]. Our understanding of protein folding has largely remained elusive due to the vast potential complexity of cooperative interactions involved in tracking such mechanisms [6-8]. Energy landscape theory provides a novel framework for understanding the global principles of protein folding in terms of funnels [9]. A greater understanding of this process can be facilitated by further insight into both the structural and dynamical changes that occur during the folding process. A combination of experiment and calculations has recently been developed to monitor these changes in tandem.

Traditionally the majority of experimental data on protein folding has been obtained through kinetics experiments which do not report on atomic level structural changes and thus the dynamics that characterize the folding process. Additionally, until recently, experimental methods with structural resolution have lacked the temporal resolution necessary to observe ultra-fast folding processes. These methods including 1DIR, florescence, NMR and XRD have resulted in indirect or time averaged information about the structure and the energy surface of proteins along the folding path. Although progress has been made by advanced NMR techniques combined with simulation lengthy folding processes have yet to be explored [9]. Recent advances in 2DIR correlation spectroscopy (2DCS), using techniques derived from NMR, are shedding new light on the mechanism of protein folding [10-14].

Similar to 1DIR absorption spectroscopy, 2DCS uses infrared wavelengths to probe the Amide-I and other vibrational bands present in protein structure [15]. The Amide-I band, consisting of the carbonyl stretch of the protein backbone is commonly probed by IR based techniques because it displays structural sensitivity due to coupling between in-phase bending of N-H and stretching of C-H bonds [16]. In proteins these bands respond to coupling between amide units and delocalization of vibrational states thus reporting on the size and secondary structure of proteins. Using advanced multiple pulse coherent spectroscopy techniques like 2DCS IR, transitions can be spread across two axes revealing vibrational couplings resulting from three-dimensional structural contacts [17-21]. Coupled with ultrafast optical techniques 2DCS IR spectroscopy results in a structure-based tool that is responsive at the ultrafast timescales present in the folding mechanism. Accordingly 2DCS IR spectroscopy is now being used to study unfolding processes in T-jump experiments.

It has been proposed that simulation of 2DCS IR spectra coupled with MD calculations can provide additional insight into the folding pathway, particularly when compared with experiment [22,23]. The accurate simulation of one-dimensional absorption spectra of proteins by incorporating a dipole-dipole coupling scheme is well established [24]. Although the simulation of 2DCS IR presents greater challenges it is now increasingly possible to reliably calculate the 2DCS IR spectra for a variety of small protein structures [21,25-28]. In these calculations a Local Amide Hamiltonian is generated with structural coordinates derived from MD simulations [28]. The signal is then simulated by a third-order response function dependent on all of the one and two-exciton states and their coupling to a thermal bath by the sum over states (SOS) method or nonlinear exciton equations (NEE). Spectra are calculated for structures, particularly intermediates, in a MD simulation of protein folding. The calculated spectra can then be compared to experimental 2DCS IR to elucidate new structural information about the folding mechanism. As increasingly accurate and tested 2DIR Hamiltonian models are produced comparison of 2D IR spectroscopy will become a viable approach to validate MD force fields [17-21,29].

**Figure 1:**
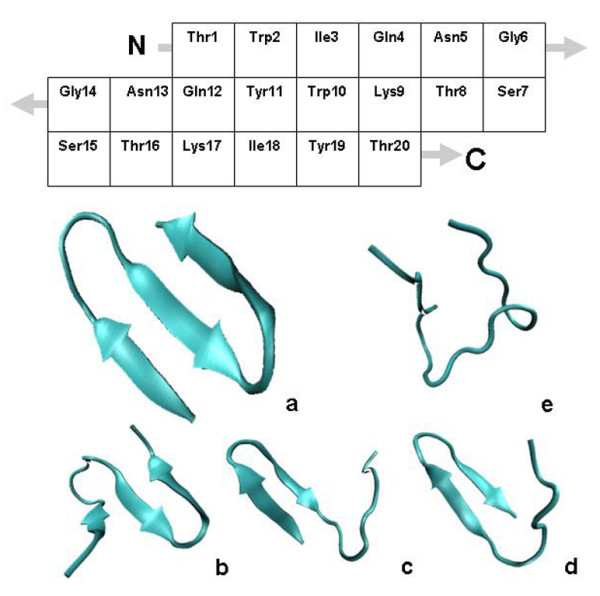
**The residue composition and investigated conformational states of Beta3s**. Top: Residues in Beta3s structure shown in native conformation. Bottom: Cartoon rendering of Beta3s in a) native b) Ns c) Cs d) Ch-Curl conformations e) 6-12 Helix.

In this study simulated 2DCS IR was used to characterize the folding mechanism of the 20 residue β-sheet mini-protein Beta3s (Figure 1). Beta3s, a de-novo three stranded β-sheet mini-protein, contains common protein structural motifs including a β-hairpin and an anti-parallel β-sheet [30,31]. The structure and folding of Beta3s has been probed extensively by NMR and through numerous computational studies, however much remains unknown about its folding mechanism [18,32-37]. This data together with the synergies of MD and 2DCS IR calculations provide a unique opportunity to reveal spectra-structure correlations and explore the folding mechanism of Beta3s from a structural perspective.

## 2. Methods

### 2.1. Beta3s Molecular Dynamics Simulations

Beta3s, a synthetic 20-residue peptide (Thr1-Trp2-Ile3-Gln4-Asn5-Gly6-Ser7-Thr8-Lys9-Trp10-Tyr11-Gln12-Asn13-Gly14-Ser15-Thr16-Lys17-Ile18-Tyr19-Thr20), [18], was folded with the CHARMM PARAM19 force field [38]. This force field explicitly models all heavy atoms and the hydrogen atoms bound to nitrogen or oxygen atoms. The folding simulations were performed with the program CHARMM [38,39], and analysis was carried out with the MMTSB [40], package as well as our own code. Solvent interactions were taken into account by an implicit model based on solvent-accessible surface area (SASA) [41]. Ten simulations of 2 ns each, starting from a linear structure and random seed were performed under default electrostatic cutoffs (7.5A) and 330 K to sample the folding pathway of Beta3s. This procedure has been used extensively by Caflisch et al. to reversibly fold Beta3s into its NMR conformation and efficiently sample its folding landscape on several occasions [32-37]. Folding to the native conformation was confirmed by fraction of native contacts (Q-score) analysis. The peptide was considered folded to the known folded NMR conformation, detailed in reference 18, when at least 25 of 26 Nuclear Overhauser Effect (NOE) constraints were satisfied, a Q-score of greater than 0.95.

### 2. 2. Conformation Identification

**Figure 2:**
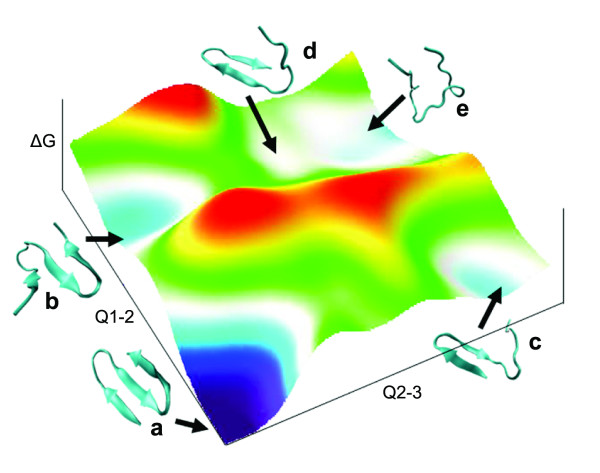
**The free energy surface of Beta3s and assigned conformational states**. Beta3s free energy surface approximated from data in previous works by Caflisch et al. [33,34]. Axis ΔG, Q1-2 and Q2-3 representing, free energy and fraction of native contacts in each of the strands N-terminal (strand 1), central, (strand 2) and C-terminal (Strand 3) respectively. Blue indicates low and red indicates high free energy values on a free energy scale from 0-4 kcal/mol. Conformations, a) native b) Ns c) Cs d) Ch-Curl conformations e) 6-12 Helix, are assigned to local basins in accordance free energies described in previous work [33,34].

Significant exploration of the conformational space of Beta3s has already been accomplished by a variety of rigorous methodologies [32-37]. (Figure 2) The most current work at the time of this study had identified several statistically significant conformations in the conformational space of Beta3s [33]. The most populated structures in the folding pathway include the mostly helical "6-12 helix" (Figure 1e), a curled structure "Ch-curl" (Figure 1d), a native like structure with the C-terminus out of register "Cs-or" (Figure 1c), a native like with the N-terminus out of register "Ns-or" (Figure 1b), and native structure (Figure 1a). These structures listed in table 1 and displayed in figure 1 were defined by Karplus and Caflisch et al. using their DSSP backbone configuration and investigated in this study [33]. (Figure 1, Table 1) The DSSP configuration identifies residue conformations based upon the φ, ψ backbone angle [42]. In our study each of the 5 conformations studies were identified by comparison of the DSSP calculated backbone configuration at each time step in the trajectories to the published backbone configurations for each intermediate. A total of five structures were chosen for each conformation investigated. Since many structures were identified and only a few were required with some heterogeneity only the middle structure of each quintile in a distribution of structures was chosen for investigation. The quintile distribution was formed from analysis of all matching structures by clustering backbone RMSD to the centroid. By this process we were able to account for slight structural variances in the backbone configurations that are more consistent with an ensemble of structures in a conformation.

**Table 1:** Conformational Properties of Beta3s

Conformation	Q	Radius of Gyration	SASA	Backbone DSSP String
612 Helix	0.59	7.98	2084	~HHHHHHHHHHHS~~~~~
Ns-or	0.99	7.45	1993	EEEESTTEEEEESSEEEE
Cs-or	0.89	7.96	2004	EEEESSEEEEESSSEEEE
Ch-Curl	0.89	7.78	1951	~~SSGGG~~~EESSEETT
Native	1.00	7.50	1783	EEEESSEEEEEESSEEEE

Averaged conformational properties of investigated Beta3s conformations, DSSP assigned backbone configuration, Q, fraction of native contacts, radius of gyration, and SASA, solvent accessible surface area.

It has been well documented that solvation and arrangement of solvent plays an important role in the 1D and 2DCS IR spectra of the Amide-I bond [26,43,44]. Consequently the identified conformations from the CHARMM PARAM19 force field were solvated and all hydrogen atoms were added. The solvation process involved an initial minimization of the solvent around a constrained protein backbone followed by 20 ps of backbone constrained molecular dynamics to allow for adjustment of the protein-water interface. The CHARMM PARAM22 [26,45], all atom force field was implemented in this process. This procedure resulted in a total of 100 unique solvent environments for the 5 unique structures of each of the 5 conformations investigated.

### 2.3. Amide I Spectral Calculations 1D and 2D IR Spectra: SPECTRON

**Figure 3:**
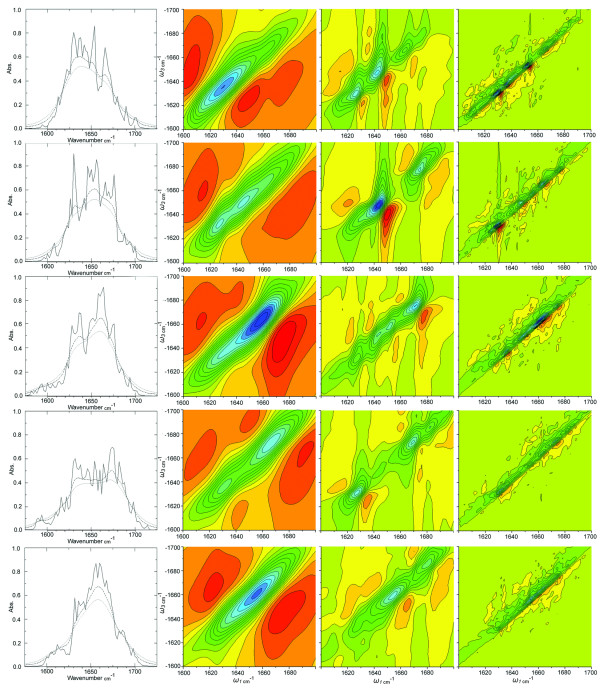
**The simulated 2DIR spectrum of Beta3s**. Right: The linear absorption spectra for investigated conformations of Beta3s calculated with homogenous broadening parameters 1 cm^-1 ^(solid line), 5 cm^-1 ^(dashed line) and 10 cm^-1 ^(dotted lines). Bottom axis is absorbance in cm^-1 ^and side axis is absorbance. a) native b) 6-12 Helix c) Cs d) Ns and e) Ch-Curl. Left: The 2DIR spectra for the **k**_I _experimental parameters for each conformation of Beta3s. Columns left to right represent line width parameters (Γ) 1 cm^-1 ^(left), 5 cm^-1 ^(middle) and 10 cm^-1 ^(right). Rows indicate data for each of the conformations top to bottom native, Ns, Cs, Ch-Curl, 6-12 Helix.

**Figure 4:**
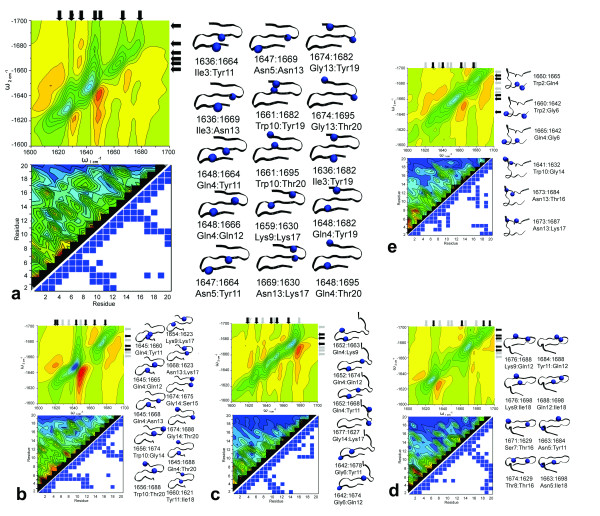
**The structure-spectral correlation for investigated conformations of Beta3s**. Letter designations a) native b) Ns c) Cs d) Ch-Curl conformations e) Helix Top Left: The 2DIR plot at Γ of 5 cm-1. Follow vertical axis arrows down until they meet horizontal axis arrows at identified peak. Black arrows highlight current conformation peaks while gray arrows highlight the native conformation peak locations. Bottom Left: The Normal Mode Decomposition plot of residue coupling intensities and structural contact map calculated at 6.7Å cutoff values. Right: The structual representation of the cross peak position and assigned residue. The blue spheres in the structure image represent the residues causing the cross peaks.

An ensemble of 500 structures for each conformation was implemented in the Amide-I spectral calculations. Simulation of the 1D and 2DCS IR spectra was carried out according to an approach described by Zhuang and Mukamel [28,46-48], as implemented in the SPECTRON [28], software package. The Local Amide Hamiltonian (LAH) approach was applied to describe the peptide structures in our calculations [49-53]. The Hamiltonian parameters were provided by Mukamel et al. as implemented in SPECTRON [28]. Vibrational couplings of different amide modes were calculated by the *ab inito *maps of Torri and Tasumi [24,54,55]. The three-pulse coherent four-wave mixing technique was simulated, where three incoming pulses with wave vectors **k**_1_, **k**_2_, **k**_3 _interact with the protein to generate a signal in the direction **k**_I_** = -k**_1_**+ k**_2_**+k**_3_. The sum over states (SOS) technique with a Lorentzian lineshape with varying FWHM values was used to simulate the 2DCS signal [28]. Details on the methods used in SPECTRON have been presented in reference 28 and 47. These parameters have been shown to provide reliable 1D and 2D spectra for both α-helical and β-content peptides that are suitable for comparison with experiment [43,47,56,57]. The **k**_I _signal is displayed in figures 3 and 4 by transforming the response function S_ν, γβα _(t_3_, t_2_, t_1_) as described in reference 47, to the frequency domain with first *t*_1 _and third *t*_3 _time delays:(1)

where *ω*_1 _and *ω*_3 _are the Fourier conjugates to the t_1 _and t_3 _respectively, t_2 _= 0.

### 2.4. Analyzing Residue Contributions Normal Mode Decomposition

Assessment of the spectra-structure correlations requires assignment of specific residues to peaks and cross peaks. Here we applied two methods, conformational difference analysis and normal mode decomposition (NMD). Conformational difference analysis coupled with two-dimensional contact maps involves qualitative examination of spectra relative to the structure of the native state and the other intermediate conformations. Since conformations have different structures the disappearance or presence of a particular peak relative to the native state and other states can reveal structure-peak correlations.

Normal mode decomposition (NMD) provides a simple analysis of the Local Amide Hamiltonian for cross peak interactions. In NMD analysis the excitonic Hamiltonian is diagonalized to obtain eigenvalues and eigenvectors for each of the residues. The eigenvalues provide a quantitative approximation of the IR frequency of each residue, although this does not fully take into account overlap between adjacent residues. The magnitude of the eigenvectors, produced for each residue-residue interaction is squared providing an approximate contribution of the residue-residue interaction for each of the 19 × 19 interactions. The strength of this coupling is displayed in the bottom left corner of figures 4a-e, stronger couplings are indicated by red peaks and weaker by blue peaks. Consequently, residue coupling contributions from non-sequential residues, a result of secondary interactions, can be observed. Further description of NMD analysis follows in the additional files included (Additional file 1).

## 3. Results and Discussion

### 3.1. 1DIR Spectra of Beta3s

The one-dimensional IR spectrum of each of the Beta3s conformational ensembles was calculated revealing significant differentiation between structures. (Figure 3) In the 1DIR, Amide-I band absorption at both high and low frequency peaks distinguish different secondary structure conformations and thus can be used to detect the degree of folding or point in the folding mechanism. Common Amide-I bands in proteins originating from the protein backbone configuration include, β structures absorbing at 1610-1640 cm^-1^(*v*_⊥_) and 1680-1690 cm^-1 ^(*v*_||_), α-helix at 1640-1650 cm^-1 ^and the 1650-1660 cm^-1 ^random coil regions [16]. The low frequency (*v*_⊥_) β absorption is a result coupling perpendicular to the β-strand while the high frequency β absorption is (*v*_||_) a result of in plane coupling [58]. The 1DIR data presented in figure 3 and table 2 has been calculated for explicitly solvated conformational ensembles with a homogenous broadening parameter (Γ) of 1.0 (solid line), 5.0 (dashed line) and 10.0 cm^-1 ^(dotted line). Experimental homogenous broadening parameters for the Beta3s structure would be expected to be around 8-10 cm^-1 ^similar to a poly-l-lysine in extended AP β-sheet conformation with measured broadening of 8 cm^-1 ^[59]. The simulation at 5 cm^-1 ^and 10 cm^-1 ^provide effects of broadening parameters that depend not only on the broadening parameter we set but also increases with the number of structures in the ensemble.

**Table 2:** 2DIR Diagonal Peak Characteristics

	Absorption Region (cm-1)				Splitting (cm-1)		
Conformation	Beta Low	Alpha Low	Alpha High	Beta High	Beta-Alpha	Alpha-Alpha	Alpha Beta
Nat	1630	1645	1665	1678	23	38	26
Ns	1638	N/A	1678	1692	42	N/A	18
Cs	1648	N/A	1678	1692	42	N/A	18
Ch	1632	N/A	1672	1688	66	N/A	16
612	1632	1662	N/A	1685	42	N/A	32

Diagonal peak characteristics calculated from 2DIR at 5 cm^-1 ^line width parameter (Γ), position and relative distance, for Beta3s conformations.

The native conformation 1DIR spectrum of Beta3s at Γ of 10 cm^-1 ^shows a maximum peak at 1636 cm^-1 ^which is consistent with the low frequency *v*_|| _mode for a β-sheet structure resulting from oscillations in phase perpendicular to the β-strands (Figure 3, Table 2) [16]. In the 10 cm^-1 ^data the dominant high frequency peak occurs at 1666 cm^-1 ^which is lower than the typical 1680 cm^-1 ^characteristic high frequency peak *v*_|| _for β-sheets. Investigation of the data obtained at Γ of 1 cm^-1 ^reveals the expected high frequency β-sheet *v*_|| _mode at 1680 cm^-1^. The data at 1 cm^-1 ^also revealed a significant peak at 1654 cm^-1^, typically associated with random coil and α-helical secondary structure [16]. The presence of the 1666 cm^-1^and 1654 cm^-1 ^peaks result from the turn region residues in the native conformation, although the absorption intensity for these modes is higher than expected. High absorption intensity is likely attributable to overlapping contributions from very similar modes occurring as a result of the structural homogeneity of the turn region versus the β-sheet regions in the ensemble. A comparison of the turn region versus β-sheet regions is described in Table S1 (see Additional file 1) and shows the β-sheet regions of Beta3s to be less homogenous in structure than the turn sections.

The major peak in the Ns conformation originated at 1653 cm^-1 ^and was surrounded by a shoulder at 1630 cm^-1 ^and another at 1671 cm^-1 ^at Γ of 10 cm^-1^. (Figure 3, Table 2) The right shoulders correspond to the high *v*_|| _frequency β-sheet absorption while the central peak and left peaks result from increased random coil character and the *v*_|| _mode of the β-sheet in this conformation [16]. Interesting, the low frequency *v*_||_, β-sheet peak shifted approximately 6 cm^-1 ^lower compared to the native state. A decreasing distance between the high and low frequency Amide-I bands of the β-sheets has been shown to correspond to decreasing β-sheet content [26]. This is expected because the Ns conformation lacks the fully formed N-terminal β-sheet, and thus contains less β-sheet content when compared to the Native state.

The Cs conformation incorporating an out of register C-terminal region contains a predominate peak at 1660 cm^-1 ^surrounded by a large shoulder at 1636 cm^-1 ^and a weak shoulder at 1676 cm^-1^. (Figure 3, Table 2) The 1DIR spectra at 1 cm^-1 ^Γ further resolves these peaks particularly the weak shoulder at 1676 cm^-1^. Relative to the Native structure a 4 cm^-1 ^decrease in width between the *v*_|| _(1636 cm^-1^) and *v*_|| _(1676 cm^-1^) modes of the β-sheet modes was observed indicative of the decrease in β-sheet structure in this conformation. Additionally, the evolution of a significant random coil peak as a result of the less structured C-terminal region was observed.

The Ch-Curl conformation is most similar to the Ns conformation containing a well ordered C-terminal β-sheet structure and disrupted N-terminal region. In the Ch-Curl structure however, the C-terminus turn is inverted. (Figure 1d) The 1DIR of Ch-Curl consists of 2 peaks at 1638 cm^-1 ^and 1671 cm^-1 ^in the 10 cm^-1 ^Γ regime, additional resolution reveals multiple strong peaks from 1630 cm^-1 ^to 1688 cm^-1^. (Figure 3, Table 2) Critical β-sheet peaks are present as expected with the well structured C-terminus in this conformation. Variation and multiple strong peaks in the range of ~1645-1660 cm^-1 ^appear to result from the mostly unstructured N-terminal part of the structure, providing the "Curl" component of this ensemble. The β-sheet peak occurred at 1638 cm^-1^, 6 cm^-1 ^higher than in the Ns conformation, likely a result of the different interactions between adjacent β-sheets due to the inversion of the C-terminus sheet [26].

The 1DIR of the 6-12 helical conformation contains a single strong peak at 1658 cm^-1 ^which corresponds the an α-helix or random coil Amide-I absorption [16]. (Figure 3, Table 2) Further resolution at Γ 1 cm^-1 ^shows a splitting of the main peak into a 1656 cm^-1 ^and 1660 cm^-1 ^peak which likely corresponds to the α-helix structure absorption from residues 6 to 12 and the remainder of the structure which is largely in a random coil configuration.

### 3.2. 2DIR Spectra Beta3s

The simulated two-dimensional IR correlation spectroscopy (2DCS) reveals three-dimensional structural information about protein structure by reporting on vibration couplings and correlations between vibrations contacts. Although 1DIR appears to be sufficient to distinguish the different conformations in the folding mechanism of Beta3s it lacks ability to reveal coupling between specific residues observed in the off-diagonal peaks of the 2D spectrum. Since 2DIR spectra are calculated with the same Local Amide Hamiltonian as the 1DIR spectra cross peak locations are identical for similar Γ parameters. The 2DIR as displayed in figure 3 was calculated for 3 different homogenous broadening parameters (Γ) of 1.0 (right), 5.0 (middle) and 10.0 cm^-1 ^(left). In figure 3 each 2DIR spectrum is split into two regions, the upper left corner (blue peaks) and lower right corner (red peaks), containing signals originating from the 0->1 and 1->2 IR transitions respectively.

The native state 2DIR spectrum of Beta3s contains a full complement of cross peak interactions of the folded protein and was used as a point of reference for the other conformations. The native state exhibited 15 distinguishable off-diagonal cross peaks in the 2DIR spectra calculated at a line width of 5 cm^-1 ^as noted in table 3 and figure 3. Three specific regions of the 2DIR spectra report on the general conformation of Beta3s, the 1620-1630 cm^-1 ^and 1650-1680 cm^-1 ^region contain signals resulting from the C-terminal β-structure. The peaks at 1636-1650 cm^-1 ^in the middle of the spectra correspond to the N-terminal sheet, while the 1675 cm^-1 ^to 1700 cm^-1 ^region from 1620-1650 cm^-1 ^reports on long-range coupling between C and N-terminal chains through the central β-sheet. The Ns 2DIR spectra contained 10 of 15 native cross peaks and a novel peak at 1621 cm^-1 ^and 1660 cm^-1 ^(Figure 4b, Table 3, 4) The Cs conformation included 4 of the native cross peaks and 2 unique signals. (Figure 4c, Table 3) The additional cross peaks were noted in the Cs conformation between the in 1642 cm^-1'^s at 1674 cm^-1 ^and 1678 cm^-1^. (Figure, 4c, Table 4) The Ch conformation exhibited the 6 of 15 native cross peak interactions likely all from the C-terminal sheet. The Ch configuration also exhibited 2 novel peaks the in 1660 cm^-1'^s at 1684 cm^-1 ^and 1698 cm^-1 ^range. (Figure, 4d, Table 3, 4) Finally, the 6-12 helix displayed no native peaks and 5 new cross peaks. (Figure 4e, Table 4)

**Table 3:** Native Peak Assignment

Assigned Peak/Residue											
***ω*_1_**	**-*ω*_3_**	**Nat**		**Ns**		**Cs**		**Ch**		**612**	

1636	1664	Ile3	Tyr11	NA	NA	NA	NA	NA	NA	NA	NA
1636	1669	Ile3	Asn13	NA	NA	NA	NA	NA	NA	NA	NA
1648	1664	Gln4	Tyr11	Gln4	Tyr11	Gln4	Lys9	NA	NA	NA	NA
1648	1666	Gln4	Gln12	Gln4	Gln12	Gln4	Gln12	NA	NA	NA	NA
1647	1664	Asn5	Tyr11	NA	NA	Gln4	Tyr11	NA	NA	NA	NA
1647	1669	Asn5	Asn13	Gln4	Asn13	NA	NA	NA	NA	NA	NA
**1661**	**1682**	**Trp10**	**Tyr19**	**Trp10**	**Gly14**	**NA**	**NA**	**Lys9**	**Gln12**	**NA**	**NA**
**1661**	**1695**	**Trp10**	**Thr20**	**Trp10**	**Thr20**	**NA**	**NA**	**Lys9**	**Ile18**	**NA**	**NA**
**1659**	**1630**	**Lys9**	**Lys17**	**Lys9**	**Lys17**	**NA**	**NA**	**Ser7**	**Thr16**	**NA**	**NA**
**1669**	**1630**	**Asn13**	**Lys17**	**Asn13**	**Lys17**	**Gly14**	**Lys17**	**Thr8**	**Thr16**	**NA**	**NA**
**1674**	**1682**	**Gly14**	**Tyr19**	**Gly14**	**Ser15**	**NA**	**NA**	**Lys9**	**Gln12**	**NA**	**NA**
**1674**	**1695**	**Gly14**	**Thr20**	**Gly14**	**Thr20**	**NA**	**NA**	**Gln12**	**Lys17**	**NA**	**NA**
											
*1636*	*1682*	*Ile3*	*Tyr19*	*NA*	*NA*	*NA*	*NA*	*NA*	*NA*	*NA*	*NA*
*1648*	*1682*	*Ile4*	*Tyr19*	*NA*	*NA*	*NA*	*NA*	*NA*	*NA*	*NA*	*NA*
*1648*	*1695*	*Ile4*	*Thr20*	*Ile4*	*Thr20*	*NA*	*NA*	*NA*	*NA*	*NA*	*NA*

Specific peak assignment of mode contributions from NMA for the native conformation of Beta3s. Non-native conformation modes noted when applicable. Mode-peak assignment for non-native conformations with slight variations in mode are presented when a peak is found in the same position as in the native conformation. Regular format table text represents modes of N-Terminal Sheet, bold table text background represents modes of C-Terminal Sheet and italic text represents modes of N- and C-terminal interaction across central sheet.

**Table 4:** Non-Native Peak Assignment

Assigned Peak/Residue							
***ω*_1_**	**-*ω*_3_**	**Ns**		***ω*_1_**	***ω*_3_**	**Cs**	

1660	1621	Tyr11	*Ile18*	1642	1678	Gly6	Lys9
				1642	1674	Gly6	Gln12

							

							

***ω*_1_**	***-ω*_3_**	**Ch**		***ω*_1_**	***-ω*_3_**	**612**	

1663	1684	Asn5	Tyr11	Trp2	Gln4	1660	1665
1663	1698	Asn5	Ile18	Trp2	Gly6	1660	1642
				Gln4	Gly6	1665	1642
				Trp11	Gly14	1641	1632
				Asn13	Thr16	1673	1684
				Asn13	Lys17	1673	1687

Specific peak assignment of mode contributions from NMA for the non-native conformation non-native peaks of Beta3s. Mode-peak assignments for non-native conformations are presented for non-native peaks.

### 3.3. Residue Contributions and Peak Assignment

One of the primary goals of 2DCS is the assignment of specific cross peaks to particular residue interactions to reveal the three-dimensional structure of proteins [15]. This has also been among the most challenging tasks for the 2DCS spectroscopists. In this work the small size of Beta3s along with the multiple conformations of known structure helps facilitate peak assignment. This is an ideal situation, unique to computation, because experimentalists normally do not have atomic conformations for comparison or work with very large proteins that complicate structure assignment and necessitate isotopic labels to isolate specific peaks [15,56].

Structure-peak assignment of Beta3s was determined by two methods, conformational difference analysis and normal mode decomposition (NMD). Examination of the Native structure ensemble alone suggests that Beta3s contains two distinct cross peak contributors originating from residues on the C-terminal and on the N-terminal β-strands. This, however, does not address which residues contribute to each peak. NMD analysis of the Native conformation of Beta3s allowed us to ascertain 15 readily identifiable residues that contribute to 15 peaks in the 2DIR spectra. (Table 3, Figure 4a) The eigenvalues from NMD allowed us to approximate the residue carbonyl group origin of peaks by assigning an IR absorption frequency to each residue. (Figure 4a, Table 3) The eigenvalues assigned to each residue by NMD are shown in Table 5. It is important to remember that NMD analysis provides absorption frequencies for each residue but that these do not fully incorporate delocalization and overlap between the modes of the residues.

**Table 5:** NMD Residue Eigenvalues

Residue	Nat	Ns	Cs	Ch	612
Thr2	1653	1646	1647	1660	1660

Ile3	1636	1640	1646	1657	1661

Gln4	1648	1645	1652	1656	1665

Asn5	1647	1644	1654	1663	1664

Gly6	1643	1642	1642	1648	1666

Ser7	1643	1650	1638	1671	1656

Thr8	1642	1640	1661	1674	1656

Lys9	1659	1654	1663	1676	1651

Trp10	1661	1656	1667	1678	1649

Tyr11	1664	1660	1668	1684	1641

Gln12	1666	1665	1674	1688	1642

Asn13	1669	1668	1674	1639	1673

Gly14	1674	1674	1677	1633	1632

Ser15	1636	1675	1632	1631	1630

Thr16	1667	1624	1631	1629	1684

Lys17	1630	1623	1627	1623	1687

Ile18	1627	1621	1684	1698	1693

Tyr19	1682	1620	1617	1613	1612

Thr20	1695	1688	1593	1594	1610

The NMD derived Eigenvalues (in frequency cm^-1^) for each residue studied in Beta3s. The conformational structure (columns 2-6) associated residue (first column) and frequency in cm^-1^. Note that NMD analysis provides frequency for each residue but that these do not fully incorporate delocalization and overlap between the modes of the residues.

NMD analysis showed 6 peaks originated from N-terminal strand residue interaction with the central strand and 6 peaks originating from C-terminal to central strand interaction. (Figure 4a, bottom left) Interestingly, NMD also revealed peaks resulting from long range coupling between C-terminal and N-terminal residues (coupled through the central strand) which provides an indicator of degree of Native structure.

NMD analysis assigns the peak at 1636 cm^-1 ^to Ile3 and a peak at 1647 cm^-1 ^to Asn5, both of which couple to central-strand Tyr11 and Asn13 at 1664 cm^-1 ^and 1669 cm^-1 ^respectively. (Table 3) This is consistent with our 1DIR data that under initial conformational analysis suggested a 1636 cm^-1 ^peak originates on the N-terminal strand. Gln4 absorbing at 1648 cm^-1 ^also interacted strongly with Tyr11 and Gln12 producing additional cross peaks at 1664 cm^-1 ^and 1666 cm^-1^. Peaks originating from Gln4 and Asn5 are established by coupling in the NMD plots in figure 4a (bottom left) but less discernable in the 2DIR spectra. These nearly identical peak locations are likely a result of strong coupling between nearby residues, significant vibrational mode delocalization. Together the peaks from 1636 cm^-1 ^to 1647 cm^-1 ^provide a spectral region representative of the degree native-ness of the N-terminal sheet. Conformational analysis supports these peaks as an indicator of the N-terminal sheet since it was also found that these peaks are not present when the N-terminal region was interrupted as occurs in the Ns conformation.

The native conformation of Beta3s exhibited 6 peaks consistent with residue interactions on the C-terminal β-strand. Specifically, Trp10 and Gly14 were found to interact with Tyr19 and Thr20 producing cross peaks from *v*_|| _interactions at 1661 cm^-1^, 1674 cm^-1 ^and 1682 cm^-1 ^and a 1695 cm^-1 ^respectively. (Figure 4a) Additionally, *v*_|| _interactions were noted between residue Lys17 at 1630 cm^-1 ^in the C-terminal sheet and Lys9 and Asn13 at 1659 cm^-1 ^at 1669 cm^-1^. Conformational analysis further supports the NMD results, since the majority of C-terminal strand residues do not produce cross peaks when interrupted in the Cs conformation. (Figures 4a)

In the Ns conformation the interactions involving Trp2 and Asn5 were not noted corresponding to the Ns out-of-register structural disruption. (Table 3, Figure 4b) Gln4 however, exhibited an interaction with residues Tyr11, Gln12 and Asn13 forming a cross peak between 1645 cm^-1 ^and 1660 cm^-1 ^as well as 1665 cm^-1 ^and 1668 cm^-1^. Residues on the C-terminal strand produced similar peaks to that of the Native structure. A blue shift of ~5 cm^-1 ^relative to the native conformation was noted for the 1656 cm^-1 ^peak resulting from coupling to Trp10 on the central β-strand. The blue shift has been observed in prior work and occurs as the Amide modes localize as a result of β-sheet unfolding [58]. The majority of the long-range interactions between the C and N-terminal β-sheets were disrupted in this conformation as anticipated. (Figure 4b, Table 3)

The Cs conformation was noted to contain similar peaks to the Native state with the exception of anticipated disruption in the C-terminal strand. (Figure 4c, Table 3) A single C-terminal interaction was noted between Gly14 and Lys17 producing the peak at 1627 cm^-1 ^and 1677 cm^-1^. Half of the N-terminal peaks were noted with peaks between Gln4 at 1652 cm^-1^and Lys9, Tyr11 and Gln12.(Figure 4c) Cross peaks at the N-terminal region as a result of Ile3 interacting with Tyr11 and Asn13 were not present in the Cs conformation. Additionally, N-terminal signals due to the Asn5 to Asn13 interaction were not noted in the spectra or by the NMD. Interestingly, new peaks evolved at 1642 cm^-1 ^and 1674 cm^-1 ^as well as 1678 cm^-1^a result of Gly6 interacting with Lys9 and Gln12 respectively. These spectral signatures reflect that structurally the N-terminal domain is perturbed in the Cs conformation as the C-terminal domain falls out of register. No blue shifts were noted relative to the native conformation in the Cs state. The contact map at the bottom of figure 4c displays the disruption of overall secondary structure. This is unsurprising, since it has been suggested that folding of Beta3s prefers to first fold a structurally stable C-terminal domain that forms a scaffolding to facilitate folding of the N-terminal domain [32-37]. In the spectra of Cs such structural changes are ultimately reflected in the red-shifting of noted peaks relative to the native state caused by the peptide backbone shifting from a β-sheet (1630-1640 cm^-1^) to a more α-helical conformation (1650-1660 cm^-1^).

The Ch-curled conformation exhibited complete disruption of the peaks corresponding to the N-terminal region. (Figure 4d, Table 3) Two additional interactions near the N-terminus indicated by peaks at 1663 cm^-1^, 1684 cm^-1 ^and 1698 cm^-1 ^attributed to Asn5 interacting with Tyr11 and Ile18. (Table 4) The remainder of the N-terminal domain did not produce cross peaks in this conformation. In the Ch-curl structure the C-terminal region is inverted relative to the native state and as such a new set of contacts causes the spectral cross peaks. The new peaks associated with the C-terminal sheet are red shifted as much as 10 cm^-1 ^but are associated with residues similar to or close to those resulting in the C-terminal peaks of the native state. (Table 3 &4) Ser7 and Thr8 interact with Thr16 at the same location as in the native state at 1629 cm^-1 ^but the cross peak is far shifted Ser7 and Thr8 locations. Largely this is a result of the conformation of residues 7 and 8 which become helical in the turn region because of N-terminal domain disruption.

Most different from the Native structure, the 6-12 helical conformation of Beta3s exhibited none of the native cross peaks. (Figure 4e, Table 3, 4) NMD revealed that although some peaks and modal interactions appear similar to those in the Native state they originate from interactions of different residues. Six new interactions were noted as described in table 4. Local backbone interactions in the 1660 cm^-1^'s between Trp2 and Gln4 and Gly6 as well as those between Asn13, Thr16 and Lys17 are typical of the random coil and α-helical structure noted here. Additionally, the NMD plot reveals significant coupled interactions along the diagonal indicating strong coupling to nearby N+1...N+3 residues [16]. (Figure 4e, bottom left) Such residue interactions are highly local and thus largely in the diagonal of the 2DIR spectra due to their very similar absorption frequencies.

### 3.4. The Folding Mechanisms

Computational studies by Caflisch et al. have suggested Beta3s folds through two possible pathways [33,34]. The main folding pathway of Beta3s starts with the formation of the C-terminal side chain contacts followed by the N-terminal contacts. (Figure 2 and Figure 4) Additionally, the reverse was also found to be possible where the N-terminal structure forms first followed by the C-terminal ones. Contacts in the turn regions were also found to form first [33,34]. The proposed folding pathways have been examined numerous times by many methodologies computationally but never experimentally [32-37]. The 2DCS IR data presented here can be coupled with 2DIR experiments to investigate the intermediates and the order in which they are sampled during the folding of Beta3s.

### 3.5. Spectral Signatures of Folding

In this study specific spectra-structure correlations have been established that can provide unambiguous indicators of conformational identity during a folding experiment. Specifically, during folding Beta3s may sample the 6-12 helical conformation on its way to the native state. Transition from the 6-12 helix conformation to a more native like structure (β-sheet) is well described by the diagonal signals which decrease from a 1660 cm^-1 ^centered primary peak to one in the 1640 cm^-1^. Moreover, evolution of folding along the primary pathway, sampling the Ns conformation before the native state, is indicated by the evolution of 1648 cm^-1 ^cross peaks a result of Gln4 coupling as well as signals from the full compliment of C-terminal peaks. (Figure 4b, Table 3) The existence of the Gln4 peaks also differentiates the Ns conformation spectrally from the Ch-Curl conformation which lacks this structure but contains a similar C-terminal spectral signature. (Figure 4d) Finally, in the alternate folding pathway the N-terminal region forms first, thus sampling the Cs conformation prior to the native state. 2DIR spectral analysis along this folding path would include the Gln4 associated cross peaks and nearly none of the C-terminal associated peaks in the 1666 cm^-1^-1680 cm^-1 ^range. (Figure 4c, Table 3)

### 3.6. Isotopic Labeling Experiments

Isotope labeling can be used to manipulate 2DCS signals by enhancing desired spectral features as demonstrated by Hochstrasser [60]. ^13^C and ^18^O labeling of peptides can induce 65 cm^-1 ^red shift of Amide-I bands providing detailed structural constraints. In this work the different conformations of Beta3s were known and so isotopic labeling is of minimal help in identifying structure-peak correlations because they can be determined by conformational analysis alone, however because the information is not available in experiments, labels may aid in tracking folding. In practice labeling of Gln4 and Tyr19 (both commercially available) could provide insight into coupling of the N-terminal sheet and C-terminal sheet respectively.

## 4. Conclusions

We have shown that 2DCS IR spectra of proteins coupled with conformational sampling though folding calculations can reveal significant structural information about the ensemble evolution in the folding mechanism. This coupled with experiment can help to provide unprecedented information about the folding process including structurally resolved folding kinetics. It is clear, even from the 1DIR, spectrum that the intermediates sampled in the folding mechanism of Beta3s exhibit distinct spectroscopic characteristics. Although 1DIR provides some insight into the specific conformation of the peptide the 2DCS method allows tracking of specific cross peaks and associated atomic contacts that provide critical indicators of the folding mechanism. The results presented here suggest that further insight into the folding pathway of Beta3s can be obtained from experimental work similar to T-jump experiments performed on Ubiquitin and Amyloids [61,62]. Additionally, considering the extent to which the Beta3s peptide has been studied computationally by a number of models, theses results can also be applied to validation of molecular dynamics force fields [32-37].

## Supplementary Material

Additional file 1 **Supplementary Data**. The data provided includes work on the validation of 2DIR protocol, Normal Mode Decomposition methodology and full native peak assignment data. Click here for file
