# Supplementary material for: Probing the folding of mini-protein Beta3s by two-dimensional infrared spectroscopy; simulation study
Source: PMC Biophys. 2010 Mar 19;3:8. doi: 10.1186/1757-5036-3-8 (PMC2851665; doi:10.1186/1757-5036-3-8)
Supplement: Additional file 1 — Supplementary Data. The data provided includes work on the validation of 2DIR protocol, Normal Mode Decomposition methodology and full native peak assignment data. [file 1757-5036-3-8-S1.PDF]

SUPPLEMENTARY MATERIALS:

Validation of 2DIR computational protocols

The computational protocols involved in the calculation of 2DIR were tested prior to this work to confirm the correct setup of the simulation software SPECTRON [1]. As a simulation software for 2DIR spectra of proteins, SPECTRON has undergone significant testing and reliable results have been produced for β-sheet containing proteins [1-4]. However, since no current 2DIR spectrum of Beta3s exists it is important to ensure our model produces reasonable results with other known systems. In this test the trpzip2 protein derived from protein databank (PDB) structure, 1LE1 [5] was equilibrated simulated by implicit solvent MD simulation, solvated and an ensemble generated according to the procedure outlined in the methods section for Beta3s. The solvation process involved an initial minimization of the solvent around a constrained protein backbone followed by 20 ps of backbone constrained molecular dynamics to allow for adjustment of the protein-water interface. The CHARMM PARAM22 [6, 7], all atom force field was implemented in this process. The ensemble was then subjected to 1D and 2DIR calculations of the Amide-I band using the k_I pulse orientation in SPECTRON. The resulting spectra produced are displayed in Supplementary Figure 1 and are in good agreement with past experimental work and theoretical calculations on trpzip2 surveyed in detail by Ganim and Tokmakoff [7] (Supplementary Figure 1). Specifically, in the 1DIR spectra the high and low frequency peak positions and relative intensities are qualitatively similar of those surveyed in Figure 3 in the Ganim and Tokmakoff review [7]. The 2DIR spectrum was also simulated and compared to previous work as shown in Supplementary Figure 1 [7]. The simulated 2DIR spectrum revealed peak shape, broadening and locations similar to those of experiment and the more accurate simulations surveyed in reference 26. The cross-peak ridge extending horizontally from the high frequency peak is observed but it is weaker than the experimental spectra. The ridge extending horizontally from the blue signal is reproduced. Finally, the node between the fundamental and overtone peaks is well represented in our model. Consequently, these results suggest that the methods we have employed in the simulation of Beta3s 2DIR are well executed and capable of providing reliable results.

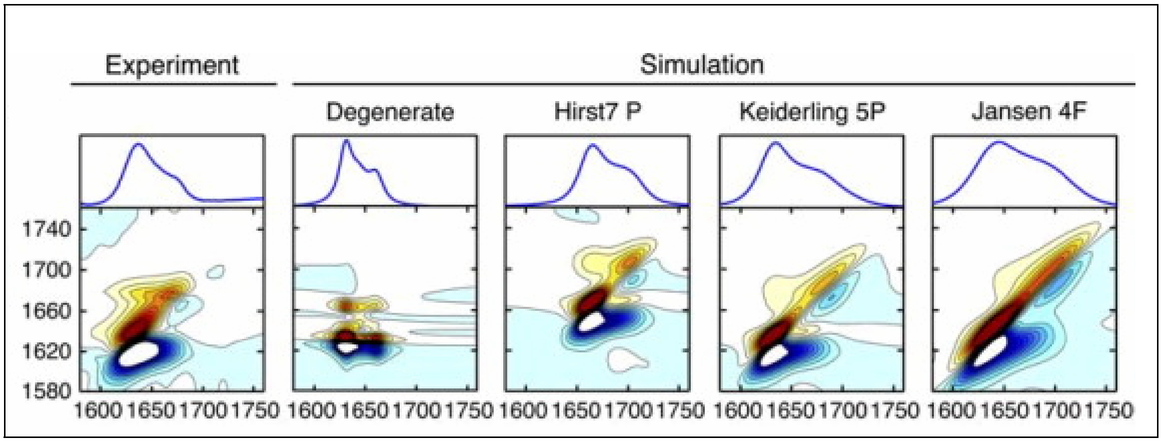

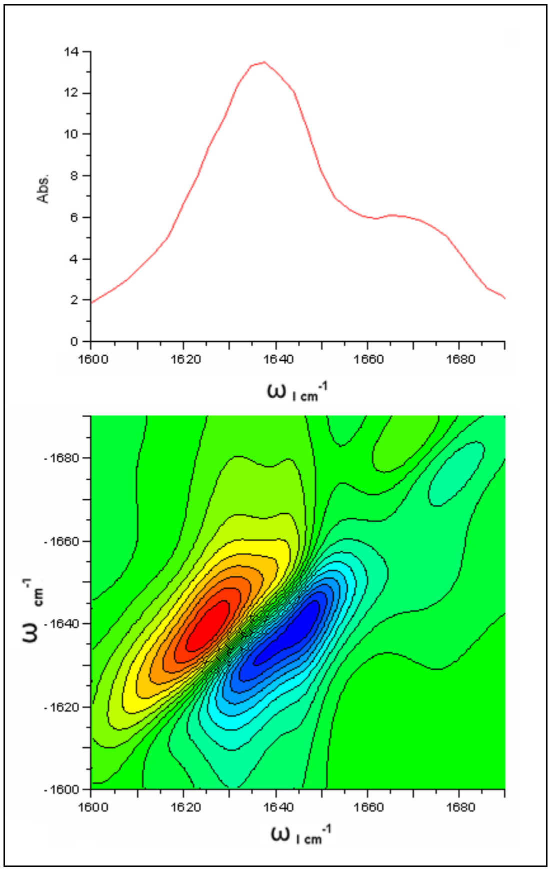

Supplementary Figure 1: Experimental and Simulated 1D and 2DIR of Trpzip2.
Top: Figure 3 of reference [7] reproduced in part with permission. The figure shows the experimental and simulated 1D and 2DIR spectra for trpzip2 for several different 2DIR Hamiltonians. Bottom: 2DIR spectra for trpzip2 as generated by SPECTRON through our procedure implemented with Beta3s. Location of major peaks and peak shapes are in good agreement with experimental spectra as well as other simulations.

Normal Mode Decomposition Method

Normal mode decomposition (NMD) is a method that provides a simple analysis of the Local Amide Hamiltonian revealing strengths of cross peak interactions and mode excitation values. In NMD analysis the excitonic Hamiltonian is diagonalized to obtain eigenvalues and eigenvectors for each of the residues in the system. The diagonalized matrix consists of Eigenvectors C_i the magnitude of which is the Eigenvalue E_i, where $HC_i=E_iC_i$. The elements in the vector C_i are can be denoted c_{ij} the wave function is represented by,

$$\left|\phi_i\right\rangle = \sum_{j=1}^{N} c_{ij} \left|\psi_j\right\rangle \qquad \text{(Supplementary Equation 1)}$$

where,

$$\sum_{j=1}^{N} c_{ij}^{\;2} = 1 \qquad \text{(Supplementary Equation 2)}$$

The Eigenvalues extracted from this diagonalization represent the frequency of each mode (residue). Consequently the c_{ij}^2 value related to the Eigenvectors represents the contribution of mode (residue) *i* to mode (residue) *j*. The contribution from each *i* and *j* is what is plotted in the NMD analysis.

Supplementary Table 1: Native Beta Region RMSD to Average

**Conformation**	**Sheet 1**	**Turn 1**	**Sheet 2**	**Turn 2**	**Sheet 3**
**Residue Range**	**1-5**	**6-8**	**9-13**	**14-16**	**17-20**
Structure 1	2.28	0.85	1.82	1.39	1.79
Structure 2	1.94	0.55	2.34	1.12	1.24
Structure 3	1.56	0.91	1.63	0.88	2.89
Structure 4	2.10	0.80	0.84	0.99	2.34
Structure 5	1.82	0.89	2.28	1.15	1.94
**Average**	**1.94**	**1.11**	**1.78**	**0.80**	**2.04**

The RMSD values for particular residue ranges for the native structure of Beta3s. The data highlights the variance in structures around the sheet regions by comparison to the turn regions.

Supplementary Table 2: Full Native Peak Assignment

ω_1	$-\omega_3$	Nat		ω_1	$-\omega_3$	Ns		ω_1	$-\omega_3$	Cs	
1636	1664	Ile3	Tyr11	NA	NA	NA	NA	NA	NA	NA	NA
1636	1669	Ile3	Asn13	NA	NA	NA	NA	NA	NA	NA	NA
1648	1664	Gln4	Tyr11	1645	1660	Gln4	Tyr11	1652	1663	Gln4	Lys9
1648	1666	Gln4	Gln12	1645	1665	Gln4	Gln12	1652	1674	Gln4	Gln12
1647	1664	Asn5	Tyr11	NA	NA	NA	NA	1652	1668	Gln4	Tyr11
1647	1669	Asn5	Asn13	1645	1668	Gln4	Asn13	NA	NA	NA	NA
1661	1682	Trp10	Tyr19	1656	1674	Trp10	Gly14	NA	NA	NA	NA
1661	1695	Trp10	Thr20	1656	1688	Trp10	Thr20	NA	NA	NA	NA
1659	1630	Lys9	Lys17	1654	1623	Lys9	Lys17	NA	NA	NA	NA
1669	1630	Asn13	Lys17	1668	1623	Asn13	Lys17	1677	1627	Gly14	Lys17
1674	1682	Gly14	Tyr19	1674	1675	Gly14	Ser15	NA	NA	NA	NA
1674	1695	Gly14	Thr20	1674	1688	Gly14	Thr20	NA	NA	NA	NA
1636	1682	Ile3	Tyr19	NA	NA	NA	NA	NA	NA	NA	NA
1648	1682	Ile4	Tyr19	NA	NA	NA	NA	NA	NA	NA	NA
1648	1695	Ile4	Thr20	1645	1688	Ile4	Thr20	NA	NA	NA	NA

ω_1	$-\omega_3$	Ch		ω_1	$-\omega_3$	612	
NA	NA	NA	NA	NA	NA	NA	NA
NA	NA	NA	NA	NA	NA	NA	NA
NA	NA	NA	NA	NA	NA	NA	NA
NA	NA	NA	NA	NA	NA	NA	NA
NA	NA	NA	NA	NA	NA	NA	NA
NA	NA	NA	NA	NA	NA	NA	NA
1676	1688	Lys9	Gln12	NA	NA	NA	NA
1676	1698	Lys9	Ile18	NA	NA	NA	NA
1671	1629	Ser7	Thr16	NA	NA	NA	NA
1674	1629	Thr8	Thr16	NA	NA	NA	NA
1684	1688	Lys9	Gln12	NA	NA	NA	NA
1688	1698	Gln12	Ile18	NA	NA	NA	NA
NA	NA	NA	NA	NA	NA	NA	NA
NA	NA	NA	NA	NA	NA	NA	NA
NA	NA	NA	NA	NA	NA	NA	NA

Supplementary Table 2)
This table highlights the exact residue contact and peak location as derived from NMD analysis. Assigned residues may differ slightly from the residues for similar peaks in the native state. This is reported here because uncertainty peak assignment due to NMD in which delocalization is not accounted for. The values were included as native like if peaks did not exceed more than a few residues away. Residues labeled with bold exhibited extreme changes from the native value to the point where they may represent peaks resulting from completely different interactions. White background represents modes of N-Terminal sheet, dark shaded background represents modes of C-Terminal sheet, light shaded region indicated peaks due to C-Terminal and N-Terminal coupling through the central sheet structure.

Supplementary References:

1. Zhuang W, Abramavicius D, Hayashi T, Mukamel S: **Simulation protocols for coherent femtosecond vibrational spectra of peptides.** *Journal of Physical Chemistry B* 2006, **110:**3362-3374.
2. Zheng J, Kwak K, Fayer MD: **Ultrafast two-dimensional IR vibrational echo spectroscopy.** *Accounts of Chemical Research* 2007, **40:**75-83.
3. Zhuang W, Abramavicius D, Voronine DV, Mukamel S: **Simulation of two-dimensional infrared spectroscopy of amyloid fibrils.** *Proc Natl Acad Sci U S A* 2007, **104:**14233-14236.
4. Abramavicius D, Zhuang W, Mukamel S: **Peptide secondary structure determination by three-pulse coherent vibrational spectroscopies: A simulation study.** *Journal of Physical Chemistry B* 2004, **108:**18034-18045.
5. Cochran AG, Skelton NJ, Starovasnik MA: **Tryptophan zippers: Stable, monomeric beta-hairpins.** *Proceedings of the National Academy of Sciences of the United States of America* 2001, **98:**5578-5583.
6. MacKerell AD, Bashford D, Bellott M, Dunbrack RL, Evanseck JD, Field MJ, Fischer S, Gao J, Guo H, Ha S, et al: **All-atom empirical potential for molecular modeling and dynamics studies of proteins.** *Journal of Physical Chemistry B* 1998, **102:**3586-3616.
7. Ganim Z, Tokmakoff A: **Spectral signatures of heterogeneous protein ensembles revealed by MD simulations of 2DIR spectra.** *Biophysical Journal* 2006, **91:**2636-2646.
